# The Herald of Health

**Published:** 1875-05

**Authors:** 


*The Herald of Health*, another valued exchange, for May, contains No. 3 of "How I Managed my Children," by Mrs. Warren; "Physiological Arguments in Favor of Vegetarianism," by Prof. Francis W. Newman; "Coughs and Colds;" "The Flower Garden and Health;" "Out of the Nettle the Flower," by Amanda T. Jones; "Hints about the Prevention of Scarlet Fever;" "How to Secure Sanitary Houses;" "Heroes of Culture," by Edwin F. Bacon; "The Liver, its Use, and How to Take Care of It," Vegetable Oils and Fats," "Studies in Hygiene," "Health of Ministers," "Choosing a Mate," "Purity for Boys," "Sunlight and Health," etc., etc. $2.00 a year, with the Complete Works of Shakespeare free, as a premium. Samples 15 cents. Address Wood & Holbrook, Publishers, 13 & 15 Laight Street, New York.

